# Retrospective Multicenter Analysis of Malignant Struma Ovarii: Clinical Characteristics, Management, and Outcomes

**DOI:** 10.3390/jcm14248807

**Published:** 2025-12-12

**Authors:** Atacem Mert Aytekin, Yagmur Arslan, Utku Akgor, Murat Cengiz, Banu Boso Aslantas, Huseyin Akilli, Cansu Turker Saricoban, Ibrahim Yalcin, Mehmet Kefeli, Onur Karaaslan, Dogan Vatansever, Ipek Betul Ozcivit Erkan, Abdullah Serdar Acikgoz, Tugan Bese, Oguzhan Kuru

**Affiliations:** 1 Division of Gynecologic Oncology, Department of Obstetrics and Gynecology, Istanbul University-Cerrahpasa, Istanbul 34153, Türkiye; atacemmert@gmail.com (A.M.A.); abdullahserdar.acikgoz@iuc.edu.tr (A.S.A.); tuganbese@gmail.com (T.B.); 2 Department of Obstetrics and Gynecology, Istanbul University-Cerrahpasa, Istanbul 34153, Türkiye; yagmur.arslan.1995@gmail.com (Y.A.); ipekbetulozcivit@gmail.com (I.B.O.E.); 3 Division of Gynecologic Oncology, Department of Obstetrics and Gynecology, Hacettepe University, Ankara 06100, Türkiye; utkuakgor@gmail.com (U.A.); drmuratcengizgyn@gmail.com (M.C.); 4 Division of Gynecologic Oncology, Department of Obstetrics and Gynecology, Baskent University Adana Hospital, Adana 01250, Türkiye; banuboso@hotmail.com; 5 Division of Gynecologic Oncology, Department of Obstetrics and Gynecology, Baskent University Ankara Hospital, Ankara 06490, Türkiye; hsynakilli@hotmail.com; 6 Department of Pathology, Istanbul University-Cerrahpasa, Istanbul 34098, Türkiye; cansu.saricoban@iuc.edu.tr; 7 Division of Gynecologic Oncology, Department of Obstetrics and Gynecology, Dokuz Eylul University, Izmir 35390, Türkiye; ibrahimyalcin73@gmail.com; 8 Division of Gynecologic Oncology, Department of Obstetrics and Gynecology, Ondokuz Mayıs University, Samsun 55139, Türkiye; 9 Department of Pathology, Ondokuz Mayıs University, Samsun 55100, Türkiye; mehmetkefeli@gmail.com; 10 Division of Gynecologic Oncology, Department of Obstetrics and Gynecology, Van Yuzuncu Yil University, Van 65080, Türkiye; onurkaraaslan62@gmail.com; 11 Division of Gynecologic Oncology, Department of Obstetrics and Gynecology, Koc University, Istanbul 34450, Türkiye; dvatansever@ku.edu.tr

**Keywords:** malignant struma ovarii, ovarian teratoma, papillary thyroid carcinoma

## Abstract

**Background/objectives:** The study aimed to present cases of malignant struma ovarii from seven centers in Türkiye and evaluate them within the context of the existing literature. **Methods:** We retrospectively analyzed clinical data from 17 patients treated at seven centers, focusing on clinical features, surgical management, pathology, thyroid function, adjuvant treatment, and outcomes. Additionally, a literature review including eight studies with 178 patients was conducted. **Results:** The mean age of patients was 44.7 years, with a mean tumor size of 9.2 cm. Elevated Ca 125 was found in 33.3% of patients, while thyroid function abnormalities and hyperthyroidism signs were rare. Pelvic pain and menstrual irregularities were common presenting symptoms. A total of 16 patients (94.1%) had unilateral tumors. Total abdominal hysterectomy with bilateral salpingo-oophorectomy and unilateral salpingo-oophorectomy were the most frequent surgical approaches. Histopathology predominantly showed classical papillary thyroid carcinoma (13 patients, 76%). All patients were FIGO stage I, with no metastasis. Thyroidectomy was performed in seven patients, identifying two concurrent thyroid cancers. Four patients received adjuvant radioactive iodine therapy. During a median follow-up of 43 months, no deaths and one recurrence were observed. The literature review showed a diagnosis age ranging 43–53 years and papillary thyroid carcinoma as the most common subtype. Thyroidectomy and RAI treatment were selectively applied. Among the reported studies, recurrence occurred in 7 of 76 patients (9.2%), while 5-year disease-free and overall survival rates exceeded 94% and 100%, respectively. BRAF mutations were uncommon. **Conclusions:** Malignant struma ovarii is a rare tumor with a favorable prognosis when diagnosed early and managed appropriately.

## 1. Introduction

Struma ovarii, is a rare ovarian teratoma composed entirely or predominantly of thyroid tissue and is classified as a monodermal teratoma [1]. Thyroid tissue is found in approximately 15% of ovarian teratomas, but struma ovarii accounts for less than 5% of all teratomas and represents only approximately 1% of all ovarian tumors [2]. For an ovarian teratoma to be classified as struma ovarii, thyroid tissue must constitute more than 50% of the total tumor mass [3]. Struma ovarii typically presents with pelvic mass and/or pelvic pain while ascites is less common and clinical or biochemical signs of hyperthyroidism are rare [4]. Although imaging findings are often nonspecific, struma ovarii appears as a heterogeneous, predominantly solid adnexal mass on ultrasound. Preoperative diagnosis of malignant struma ovarii (MSO) is particularly challenging due to its vague symptoms and nonspecific imaging characteristics, with histopathology required for confirmation. Thyroid function tests (thyroid stimulating hormone (TSH), free triiodothyronine (fT3), and free thyroxine (fT4)) are usually normal, though occasional abnormalities may suggest functional thyroid tissue. Tumor markers such as cancer antigen 125 (Ca 125), Ca 19-9, human chorionic gonadotropin (β-hCG), and alpha-fetoprotein (AFP) are typically nonspecific or within normal limits [5].

Germ cell ovarian tumors are typically unilateral and diagnosed at an early stage, most often stage IA, and are staged using the International Federation of Gynecology and Obstetrics (FIGO) system for epithelial ovarian cancer [6]. MSO, a rare variant accounting for approximately 5–10% of all struma ovarii cases, shares similar characteristics—usually being diagnosed at an early stage, most often stage I. The most common histological subtype is papillary thyroid carcinoma (PTC), followed by follicular thyroid carcinoma [7,8]. Metastasis is rare in MSO [9].

MSO generally has a favorable prognosis, but its optimal management remains uncertain due to limited data. While oophorectomy is usually sufficient for benign cases, treatment of MSO may require additional interventions such as thyroidectomy and radioactive iodine treatment (RAI) (I131) [10]. Currently, there are no standardized guidelines for adjuvant treatment in the postoperative period or long-term follow-up, primarily due to the rarity of the disease. Given the scarcity of published clinical outcomes, we aimed to present cases of MSO from centers in Türkiye and evaluate their management and follow-up in the context of the existing literature.

## 2. Materials and Methods

Following the institutional ethics committee approval (Approval Date: 31 October 2024, Approval Number: 1133792) and written informed consents, MSO cases were retrospectively collected from 7 centers in Türkiye based on pathology reports of ovarian specimens between 2011 and 2024. The electronic medical records of the patients were retrospectively analyzed and all data, including demographic features, preoperative evaluation findings such as biochemical parameters, treatment modalities, follow-up information, and survival outcomes were systematically collected.

The reference intervals of the biochemical parameters used in this study were determined according to the manufacturers’ manuals of the relevant tests and the current literature information: Ca 125 < 35 U/mL, Ca 19-9 < 39 U/mL, thyroid stimulating hormone TSH 0.27–4.2 μU/mL, fT3 2–4.4 pg/mL, fT4 0.93–1.7 ng/mL, AFP < 13 U/mL, β-hCG < 5 mU/mL, thyroglobulin (Tg) 0–40 ng/mL, and thyroglobulin antibody (TgAb) 0–115 U/mL.

The stages of struma ovarii were reported according to the updated 2021 FIGO staging for ovarian cancer [6]. Disease-free survival (DFS) refers to the period following successful treatment during which the patient remains free of disease-related signs or symptoms [11]. Overall survival (OS) is measured from the time of diagnosis to either the last follow-up or the patient’s death [12].

### 2.1. Literature Search

A comprehensive review of the current literature was conducted to evaluate the clinical characteristics and optimal management strategies for MSO. The search was performed in the PubMed/MEDLINE database up to May 2025. The search was performed using the following terms interchangeably: “malignant struma ovarii,” “struma ovarii with malignant transformation,” “struma ovarii papillary thyroid carcinoma,” “struma ovarii follicular thyroid carcinoma,” and “struma ovarii with concurrent primary thyroid carcinoma”. We included case series with more than five patients. Case reports, letters to the editor, abstract-only publications, and studies without surgical pathological confirmation were excluded. Additionally, studies for which full-text access was not available online—especially older publications—were also excluded. Based on these criteria, a total of eight English-language studies were included in this review.

### 2.2. Pathological Diagnosis

Histopathological evaluation was performed in accordance with the 2020 WHO Classification of Female Genital Tumours and the WHO Classification of Thyroid Tumours [13,14]. Struma ovarii was defined as a monodermal teratoma in which thyroid tissue composed more than 50% of the tumor. MSO was diagnosed when a thyroid-type carcinoma arose within struma ovarii. PTC was identified based on the required nuclear features, including enlarged overlapping nuclei, optically clear chromatin (“Orphan Annie eye”), nuclear grooves, and intranuclear pseudoinclusions. Architectural patterns such as papillary, follicular, or mixed forms were recorded. Follicular thyroid carcinoma was diagnosed only in the presence of unequivocal capsular and/or vascular invasion, following the WHO 2020 strict criteria. Poorly differentiated thyroid carcinoma was defined using the WHO criteria (solid/trabecular/insular growth pattern, ≥3 mitoses per 10 HPF, tumor necrosis, and convoluted nuclei). Strumal carcinoid was diagnosed when mature thyroid tissue coexisted with a neuroendocrine tumor component showing positive neuroendocrine markers. All diagnoses were made by experienced gynecologic pathologists from participating centers.

### 2.3. Statistical Analysis

Statistical analysis was performed using SPSS 28.0 software. The normality of distribution for continuous variables was assessed using the Shapiro–Wilk test. Descriptive statistics were reported as mean ± standard deviation (SD) for normally distributed variables and as median (minimum-maximum) for non-normally distributed continuous variables. Descriptive statistics for categorical variables were presented using frequencies and percentages. Survival analysis was performed using the Kaplan–Meier methods.

## 3. Results

### 3.1. Results of Our Case Series

Data from 17 patients across seven centers were analyzed. The mean age was 44.65 ± 15.76 years, and the mean body mass index (BMI) was 28.97 ± 4.11 kg/m^2^; three patients were nulliparous. Preoperatively, serum β-hCG and AFP levels were within normal limits in all evaluated patients (10/10 and 5/5, respectively). Ca 125 was measured in 15 patients and was elevated in 5 (33.3%), whereas Ca 19-9 was measured in 11 patients and was elevated in 2 (18.2%). Thyroid function tests were mostly normal; no patients showed clinical hyperthyroidism (Table 1). Among 15 patients with symptom data, pelvic pain was reported in 7 (46.7%), menstrual irregularities in 6 (50%), and ascites in 3 (20%). The preoperative imaging findings were unavailable.

Sixteen patients (94%) underwent surgery for adnexal mass and one during a cesarean section. The mean tumor size was 9.17 ± 3.93 cm (4–15). The tumors were unilateral in 16 of 17 patients (94.1%) and bilateral in 1 patient (5.9%). Surgical approaches included total abdominal hysterectomy with bilateral salpingo-oophorectomy in six patients (35.3%), unilateral salpingo-oophorectomy in four patients (23.5%), laparoscopic ovarian cystectomy in three patients (17.6%), debulking surgery in three patients (17.6%), and fertility-sparing debulking surgery in one patient (5.9%). Of the four salpingo-oophorectomies, one was performed during a cesarean section, while the remaining were performed laparoscopically. In the patient who underwent fertility-sparing debulking, appendectomy and omentectomy were also performed. Intraoperative frozen sections (*n* = 10) yielded benign results in six (60%) patients and malignant results in four (40%) patients (Table 2). Final pathology showed classical PTC in 13 patients (76.4%), follicular variant of papillary thyroid carcinoma in 2 patients (11.8%), poorly differentiated thyroid carcinoma in 1 patient (5.9%), and strumal carcinoid in 1 patient (5.9%). Three patients who initially underwent cystectomy later required completion surgery; however, ascites cytology remained negative for malignant cells. All patients were FIGO stage I with no metastasis; BRAF mutations were absent in all tested cases (*n* = 6). In seven patients, Tgs and TgAbs were evaluated; Tg was elevated in two patients and TgAb was elevated in one patient.

Thyroidectomy was performed in seven patients, two of whom had concurrent papillary thyroid microcarcinoma (Table 2). Four patients received RAI therapy. Two had concurrent papillary thyroid microcarcinoma identified in the thyroidectomy specimens. One patient had PTC with a focal anaplastic component within the ovarian tumor, representing a high-risk histological feature. The remaining patient had a large ovarian tumor with extensive PTC components. All patients received RAI based on thyroidectomy findings, high-risk histopathological features, and multidisciplinary team recommendations (gynecologic oncology + endocrinology). During follow-up, TgAb levels were evaluated in five patients at 6 months and in four patients at 12 months, revealing all normal values. TSH levels were assessed in ten patients at 6 months postoperatively, among whom only one patient’s levels were elevated. At 12 months, TSH was measured in eight patients, revealing low levels in one patient and elevated levels in two. Levothyroxine was given to six patients.

Median follow-up was 43 months (10–163). No deaths were recorded. Median DFS was not reached. The mean DFS (153.82 ± 8.90 months) (Mean Survival Time ± Standard Error) represents a Kaplan–Meier survival estimate. One patient, initially treated with salpingo-oophorectomy during a cesarean section and diagnosed with PTC with focal anaplastic carcinoma, had no malignancy in the thyroidectomy specimen. Six months postoperatively, while on adjuvant RAI, recurrence occurred at the thoracic vertebra and was treated with radiotherapy, with no further recurrence over 68 months of follow-up.

### 3.2. Results of the Literature Review

Eight English-language studies comprising 178 patients, including our cases, were reviewed (Table 3). Mean/median age ranged 43–53 years. Based on the available studies, signs of hyperthyroidism were reported in 5 of 84 (6%) patients with MSO. Presenting symptoms ranged from asymptomatic findings to pelvic pain or abdominal mass. Tumor size varied between 0.8 and 13.5 cm, and most tumors were unilateral. USO was the most common gynecologic surgery performed, and PTC was the predominant histopathological subtype, followed by FV-PTC. Among the five studies reporting thyroid surgery (*n* = 121), thyroidectomy was performed in 22 patients (18.2%) and RAI therapy was administered to 13 patients (10.7%). Across six studies reporting recurrence (*n* = 76), recurrence occurred in 7 patients (9.2%), while death was documented in 7 of 134 patients (5.2%). Despite limited data, five-year DFS exceeded 94.1% and OS reached 100%, with follow-up durations ranging from 24 to 96 months. BRAF mutation testing was reported in three studies (21 patients), among whom 4 were positive for the BRAFV600E mutation, all from the Schmidt et al. series [15].

## 4. Discussion

This multicenter study represents one of the largest series on MSO, a rare entity with <200 cases reported in the literature. Struma ovarii, the most common monodermal teratomas, may rarely undergo malignant transformation, most often to PTC [7,19]. In our cohort, PTC was the predominant subtype. Consistent with prior reports, thyroid function was usually normal, and hyperthyroidism was absent. Most patients underwent extensive surgery due to large tumors, though fertility-sparing procedures were selectively performed. Clinical presentation was variable, with pelvic pain and menstrual irregularities being the most common. Ca 125 elevation and ascites were also observed, but these markers lacked diagnostic specificity.

MSO typically presents with nonspecific clinical findings, making preoperative diagnosis challenging due to its resemblance to other ovarian malignancies and the frequent absence of hyperthyroid symptoms. Devaney et al. reported no distant metastasis in their 13-patient series, with clinical hyperthyroidism observed in only one case [8]. Similarly, all patients in our study were staged as FIGO stage I and showed no evidence of metastatic disease or clinical signs of hyperthyroidism. In fact, hyperthyroidism has been reported in only 8% of patients with MSO [20]. Consistent with previous studies, the most common presenting symptoms in our study were pelvic pain and menstrual irregularities [16,21]. Patients may also present with an adnexal mass and ascites, a clinical picture known as Pseudo-Meigs’ syndrome. Although Ca 125 levels may be elevated, they are not reliable for distinguishing between benign and MSO. In our study, elevated Ca 125 was observed in 10 of 15 patients (66%), and intra-abdominal ascites was noted in 3 of 15 patients (20%), findings that align with prior reports [22,23]. In addition to this, in our study, the mean age of the patients was 44.6 years, consistent with the literature which reports 40–60 [24].

Synchronous primary thyroid carcinoma in patients with MSO accounts for approximately 5–10% of cases and is significantly more common than ovarian metastases originating from a primary thyroid malignancy [25]. It is significantly more common than ovarian metastases originating from a primary thyroid malignancy, which are extremely rare [26,27]. In our study, synchronous papillary thyroid microcarcinoma was identified in two patients. As noted in previous studies, these thyroid malignancies were determined to be independent primary cancers rather than metastases [28]. In a large study by Ayhan et al. including 178 patients, synchronous thyroid carcinoma was identified in 19 patients (10.7%), and among the 72 patients who underwent thyroidectomy, 15 had synchronous thyroid cancer (20.9%) [29]. In comparison, Siegel et al. reported synchronous thyroid carcinoma in 1 of 16 patients (6.2%), and 3 of 14 patients who underwent thyroidectomy (21.4%) had synchronous disease [30]. In our study, synchronous papillary thyroid microcarcinoma was detected in 2 of 17 patients (11.7%) and in 2 of 7 patients who underwent thyroidectomy (28.5%). Although this higher percentage may be influenced by our relatively small sample size, it still supports the trend observed in the literature. These findings highlight the need for careful postoperative evaluation, case-by-case consideration of thyroidectomy, and stronger multidisciplinary management, including endocrinology input. However, a recent 15-case series reported that only 53.3% of patients received endocrinology input, highlighting a significant gap in multidisciplinary care [18].

Intraoperative frozen section is commonly used for adnexal masses; however, its diagnostic accuracy for MSO remains limited. It is often misdiagnosed as mature teratoma [31], as seen in our study, where only 4 of 10 cases were correctly identified intraoperatively. Reported cases, including ours, highlight frequent discrepancies between frozen section and final histopathology [32,33]. The combination of nonspecific clinical signs, inconclusive imaging findings, and discordance between frozen section and final pathology results makes intraoperative decision-making particularly challenging.

BRAF and RAS mutations have been reported in MSO, though their clinical significance is unclear. Schmidt et al. found BRAF mutations in four of six MSO cases but none in all nine benign cases, suggesting that malignant transformation in struma ovarii may follow molecular pathways similar to those in PTC [34]. In contrast, Shuanzeng Wei et al. detected no BRAF mutations in any of the nine MSO cases they analyzed, eight of which were of the follicular variant. The authors suggested that the lack of BRAF mutations might be attributed to the histological subtype [3]. Similarly, in our study, none of the six patients tested were found to have BRAF mutations. In PTC, BRAF mutations are observed in approximately 30–65% of cases and are associated with more aggressive behavior and poorer prognosis [35]. While the prognostic role of BRAF in MSO remains uncertain, its presence may suggest potential for aggressive clinical behavior, meriting further study.

Previous studies on MSO report excellent long-term outcomes. In a 2015 study by Goffredo et al., OS rates at 5, 10, and 20 years were 96.7%, 94.3%, and 84.9%, respectively [17]. Similarly, Sijian Li et al., in a cohort of 194 patients, reported OS rates of 91.4%, 87.7%, and 83.5% at 5, 10, and 15 years, respectively, while DFS rates were 93.8%, 90%, and 85.7%. That study also highlighted that stage IV disease was significantly associated with reduced DFS [36]. In another study, the 5-year progression-free survival (PFS) and OS were reported as 72.5% and 91%, respectively [29]. In our cohort, with all stage I patients and a median follow-up of 43 months, no deaths occurred, the 5-year DFS was 94.1%, and only one recurrence was observed. Despite the small sample and limited follow-up, our findings support the favorable prognosis of MSO.

Based on our multicenter experience and the literature, patient selection for thyroidectomy and RAI therapy should be individualized. Thyroidectomy may be considered in patients with synchronous thyroid carcinoma or suspicious thyroid nodules, aggressive ovarian histology (poorly differentiated carcinoma, capsular/vascular invasion, or anaplastic components), extra-ovarian spread, large tumors (>4–5 cm), or elevated postoperative Tg/TgAb levels. In contrast, patients with stage I disease, classical or follicular variant PTC confined to the ovary, and normal postoperative thyroid markers may be safely managed with ovarian surgery alone. Thus, the role of thyroidectomy and RAI should be tailored to individual risk profiles rather than routinely applied.

This study is limited by its retrospective design, incomplete clinical data, and small sample size due to the rarity of the disease. Additionally, we did not perform a systematic review and therefore excluded case reports or case series with fewer than five patients to minimize heterogeneity; however, we acknowledge that more comprehensive data are needed. While central pathological reanalysis was not performed, diagnoses were standardized across centers by experienced gyne-oncological pathologists. But importantly, our pathological classification strictly followed the WHO 2020 criteria, which ensured standardized reporting across all seven centers. Further research is needed to clarify optimal management, treatment outcomes, and associated morbidity and mortality.

## 5. Conclusions

In conclusion, MSO is a rare ovarian tumor with generally favorable prognosis, but diagnosis, treatment, and follow-up strategies remain unclear. Surgery remains the mainstay of treatment, yet the extent of surgery, role of total thyroidectomy, and need for adjuvant therapies like RAI or levothyroxine are not standardized. Multidisciplinary management, including endocrinology input, is crucial, especially to detect possible synchronous thyroid carcinoma. In select cases, fertility-sparing surgery may be considered, emphasizing individualized treatment planning.

## Figures and Tables

**Table 1:** Demographic and clinical characteristics.

Variables		N	Mean ± SD or Median (Min–Max) or *n* (%)
Age (years)		17	44.65 ± 15.76
Parity		16	2.38 ± 1.96
BMI (kg/m^2^)		13	28.97 ± 4.11
Ca 125 (U/mL)		15	24.00 (3.37–11.27)
Ca 19-9 (U/mL)		11	16.00 (2.00–64.00)
TSH *(*μU/mL)		13	1.43 (0.0015–3.90)
Free T3 (pg/mL)		7	3.19 (2.24–7.68)
Free T4 (ng/mL)		9	9.48 (0.88–20.13)
AFP (u/mL)		6	2.23 (1.23–5.60)
Beta-hCG (mu/mL)		10	1.00 (0.1–2.4)
Presenting Complaints	Ascites	15	3 (20%)
	Pelvic pain	15	7 (46.7%)
	Menstrual irregularity	12	6 (50%)

BMI: body mass index, Ca 125: cancer antigen 125, Ca 19-9: cancer antigen 19-9, TSH: thyroid stimulating hormone, AFP: alpha-fetoprotein, Beta-HCG: human chorionic gonadotropin, Min: minimum, Max: maximum, SD: standard deviation.

**Table 2:** Frozen and final histopathological results of the patients.

**Frozen Section Results** ***n* (%)** **(N = 10)**		
Benign (*n* = 6)	Mature cystic teratoma	3 (30%)
	Struma ovarii	2 (20%)
	Benign cystic lesion	1 (10%)
Malignant (*n* = 4)	Malignant struma ovarii	1 (10%)
	Sex cord–stromal tumor	1 (10%)
	Granulosa cell tumor	1 (10%)
	Follicular variant papillary thyroid carcinoma	1 (10%)
**Final histopathological results** ***n* (%)** **(N = 17)**		
Classical variant of papillary thyroid carcinoma		13 (76.4%)
Follicular variant of papillary thyroid carcinoma		2 (11.8%)
Poorly differentiated thyroid carcinoma		1 (5.9%)
Strumal carcinoid		1 (5.9%)
**Thyroidectomy histopathological results** ***n* (%)** **(N = 7)**		
Nodular hyperplasia		1 (14.3%)
Colloid nodular goiter		1 (14.3%)
Multinodular goiter		3 (42.9%)
Papillary microcarcinoma		2 (28.5%)

**Table 3:** Summary of case series in the literature on malignant struma ovarii.

Author, Year	Country	Sample Size (N)	Mean or Median Age at Diagnosis (year)	Signs of Hyperthyroidism (*n*, %)	Most Common Presenting Symptom	Mean or Median Tumor Size (cm)	Most Frequent Tumor Laterality and Gynecological Surgery	Most Common HISTOPATHOLOGIC Subtype	TS (*n*, %)	RAI (*n*, %)	Recurrence (*n*, %)	Death (*n*, %)	5-Year DFS (%)	5-Year OS (%)	Mean or Median Follow-Up Duration (Months)	BRAF Mutation (*n*, %)
Devaney et al., 1993 [8]	USA	13	50	1, 7.7%	Mass lesion	10.7	Unilateral USO	PTC	N/A	N/A	2, 15.4%	1, 7.7% ^1^	N/A	N/A	87.6	N/A
Schmidt et al., 2007 [15]	USA	6	47	N/A	N/A	N/A	Unilateral N/A	FV-PTC	N/A	N/A	N/A	N/A	N/A	N/A	N/A	4, 66.7%
Garg et al., 2009 [9]	USA	10	44.5	N/A	Pelvic pain	0.8	Unilateral USO	FV-PTC	2, 20%	2, 20%	2, 20%	0	N/A	N/A	41.3	N/A
Robboy et al., 2009 [16]	USA	28	46.3	4, 14%	Abdominal swelling, pain or mass	13.5	Unilateral N/A	PTC	N/A	N/A	N/A	N/A	N/A	N/A	N/A	N/A
Wei et al., 2015 [3]	USA	10	53	N/A	N/A	N/A	N/A N/A	FV-PTC	N/A	N/A	1, 10%	N/A	N/A	N/A	N/A	0
Goffredo et al., 2015 [17]	Canada	68	43	N/A	N/A	5.28	Unilateral USO	N/A	6, 8.8%	3, 4.4%	N/A	6, 8.8% ^2^	N/A	96.7	96	N/A
Addley et al., 2021 [2]	England	11	48.9	0	Asymptomatic	N/A	N/A Cystectomy-BSO	PTC	3, 27.2%	3, 27.2%	0	0	100	100	24 (median)- N/A	N/A
Ryu et al., 2023 [18]	Korea	15	48	0	No symptom/incidental finding	3.3	Unilateral TH BSO	PTC	4, 26.7%	1, 6.7%	1, 6.7%	0	N/A	N/A	33 (median)	N/A
Aytekin et al., 2025	Türkiye	17	44	0	Pelvic pain	9.17	Unilateral TH BSO	PTC	7, 41%	4, 23%	1, 5.9%	0	94.1	N/A	43 (median)	0 (0/6)

^1^ One patient died due to congestive heart failure. ^2^ One patient died from malignant struma ovarii, two from heart disease, one from kidney disease, one from another type of cancer, and one from unspecified causes. BSO: bilateral salpingo-oophorectomy, DFS: disease-free survival, FV-PTC: follicular variant of papillary thyroid carcinoma, N/A: not available, OS: overall survival, PTC: papillary thyroid carcinoma, PTMC: papillary thyroid microcarcinoma, RAI: radioactive iodine therapy, TH: total hysterectomy, TS: thyroid surgery, USO: unilateral salpingo-oophorectomy.

## Data Availability

Data is contained within the article.
